# Comment on “The Origin of Surface Energy of Calcite and Its Wettability”

**DOI:** 10.1021/acs.langmuir.6c00853

**Published:** 2026-07-02

**Authors:** M. Bruno

**Affiliations:** Dipartimento di Scienze della Terra, 9314 Università degli Studi di Torino, Via Valperga Caluso 35, Torino I-10125, Italy

Recently, an article was published in Langmuir^1^ where empirical molecular dynamic (MD) calculations were performed to establish a quantitative relationship for surface broken bond density (*D*_*b*_) and interlayer spacing (*d*) on the surface energy of dry crystal faces of calcite (CaCO_3_). This relationship was obtained by calculating the surface energy, by means of MD simulations at *T* = 298.15 K, of the following crystal surfaces: (10.4), (01.8), (00.1), (21.4), (10.0), and (01.2). Moreover, the effects of water coverage and salinity on the surface energy of the (10.4) face of calcite were explored, indicating a negative relationship between water coverage and surface energy.

After carefully reading this article, we believe that the results presented in it are affected by two serious approximations, which are detailed below:

1. The (00.1) and (01.2) surfaces are dipolar ones:^2,3^ type III surfaces, according to Tasker’s classification.^4^ The type III surface is charged and there is a perpendicular dipole moment. These surfaces have infinite surface energies (or very large surface energies for finite crystals) and produce a polarizing electric field in the bulk. An electrostatic argument therefore indicates that such surfaces cannot exist. Indeed, by increasing the thickness of the slab used to simulate the crystal surface, the electrostatic potential *V* at a point above the crystal surface diverges (*V* → ∞). A more detailed discussion is reported in the paper by Hartman.^5^

In order to perform calculations on these surfaces, it is mandatory to cancel out the dipole moment by means of “surface reconstruction”. The reconstruction must be performed by modifying the density of ions in the outermost layers of the crystal by the introduction of vacancies. In particular, two reconstructions of the (00.1) face are possible (i.e., R1: 50% reconstruction; R2: octopolar reconstruction), as described in a previous paper,^3^ with different surface energies associated. But, the authors do not specify which reconstruction was considered in their calculations and it is not possible to determine this by looking at Figure 2.

Furthermore, there are two possible surface terminations for both (00.1) and (01.2) faces of calcite (i.e., Ca-terminated and CO_3_-terminated).^2,3^ Again, different surface terminations imply different surface energies, but the authors did not consider all possible surface terminations.

To further detail the importance of surface terminations and reconstruction on surface energy values, the surface energy values for the different terminations and reconstruction of the (00.1) face of calcite, as calculated by Bruno et al.,^3^ are (00.1)_*R*1_^Ca^ = 0.834, ${\left(00.1\right)}_{R1}^{{CO}_{3}}$ = 0.764, (00.1)_*R*2_^Ca^ = 0.849 and ${\left(00.1\right)}_{R2}^{{CO}_{3}}$ = 0.711 J/m^2^. It is therefore evident that different surface reconstructions and terminations lead to rather different results. Unfortunately, Liu et al.^1^ do not provide a detailed analysis of the (00.1) and (01.2) surfaces.

2. The relation used to calculate the wet surface energy, γ_*wet*_, is thermodynamically inconsistent. This means that all the γ_*wet*_ values listed in the paper (e.g., Figure 6) are ambiguous and not usable in thermodynamics. The eq 4 used by the authors is the following:

$$\gamma_{wet}=\gamma_{dry}+\frac{\left(E_{hyd}-E_{surface}-E_{H_{2}O}\right)}{2S}$$

where γ_*dry*_ is the surface energy of the crystal face in a vacuum and the quantity $\frac{\left(E_{hyd}-E_{surface}-E_{H_{2}O}\right)}{2S}$ is the adsorption energy of water (β_*ads*_) on the crystal surface with its sign reversed:^6^

$$\beta_{ads}=-\frac{\left(E_{hyd}-E_{surface}-E_{H_{2}O}\right)}{2S}$$

The correct equation to use to calculate γ_*wet*_ is the following:

$$\gamma_{wet}=\gamma_{dry}+\gamma_{water}+\frac{\left(E_{hyd}-E_{surface}-E_{H_{2}O}\right)}{2S}$$

where γ_*water*_ (0.073 J/m^2^)^7^ is the surface tension of water at *T* = 300 K. Indeed, γ_*wet*_ is the specific energy of the interface crystal/water, so it is an interface energy. It is well-known from the Dupré relation^8^ that the interface energy of a crystal/water interface (A = crystal and B = water) is given by

$$\gamma_{A/B}=\gamma_{A}+\gamma_{B}-\beta_{ads}=\gamma_{A}+\gamma_{B}+\frac{\left(E_{hyd}-E_{surface}-E_{H_{2}O}\right)}{2S}$$

where γ_A/B_ is the interface energy of the A/B interface (that is γ_*wet*_ for Liu et al.^1^), γ_A_ is the surface energy of the crystal (that is γ_*dry*_ for Liu et al.^1^) and γ_B_ is the surface tension of water (γ_*water*_). The term γ_B_ = γ_*water*_ is lacking in the eq 4 of Liu et al.^1^ This means that it is needed to add the quantity γ_*water*_ (0.073 J/m^2^) to all the γ_*wet*_ values reported in the paper. For example, the incorrect γ_*wet*_ values for the (10.4) calcite surface calculated by Liu et al.^1^ are 0.492, 0.408, 0.323, and 0.246 J/m^2^ at H_2_O coverages of 25%, 50%, 75%, and 100%, respectively. The correct values are instead 0.565, 0.481, 0.396, and 0.319 at H_2_O coverages of 25%, 50%, 75%, and 100%, respectively: the trend of the surface energy values is the same but the absolute values are different.

The “true surface energy” γ_*wet*_ must be calculated by means of Dupré’s relation, because it is the one that correctly defines the crystal/solution interface energy: it is the one used, e.g., to determine the equilibrium morphology of a crystal and to estimate the activation energy for crystal nucleation.^8^ The values of γ_*wet*_ calculated with eq 4 by the authors cannot be used in any thermodynamic relation without adding the term γ_*water*_ (0.073 J/m^2^).

A minor mistake in the paper by Liu et al.^1^ is that eq 2, p 174, is not correct, it is written with the signs reversed. The correct one is

$$\gamma_{dry}=\frac{\left(E_{surface}-E_{bulk}\right)}{2S}$$

But this is probably just an oversight, because the energy values of dry surfaces turn out to be positive numbers, as they always should be.
